# How miR-31-5p and miR-33a-5p Regulates SP1/CX43 Expression in Osteoarthritis Disease: Preliminary Insights

**DOI:** 10.3390/ijms22052471

**Published:** 2021-02-28

**Authors:** Viviana Costa, Marcello De Fine, Valeria Carina, Alice Conigliaro, Lavinia Raimondi, Angela De Luca, Daniele Bellavia, Francesca Salamanna, Riccardo Alessandro, Giovanni Pignatti, Milena Fini, Gianluca Giavaresi

**Affiliations:** 1 SC Scienze e Tecnologie Chirurgiche-SS Piattaforma Scienze Omiche per Ortopedia Personalizzata, IRCCS Istituto Ortopedico Rizzoli, 40136 Bologna, Italy; valeria.carina@ior.it (V.C.); lavinia.raimondi@ior.it (L.R.); angela.deluca@ior.it (A.D.L.); daniele.bellavia@ior.it (D.B.); francesca.salamanna@ior.it (F.S.); milena.fini@ior.it (M.F.); gianluca.giavaresi@ior.it (G.G.); 2 IRCCS Istituto Ortopedico Rizzoli, 40136 Bologna, Italy; marcello.define@gmail.com (M.D.F.); giovanni.pignatti@ior.it (G.P.); 3 Department of Biomedicine, Neuroscience and Advanced Diagnostics (Bi.N.D.), Section of Biology and Genetics, University of Palermo, 90133 Palermo, Italy; alice.conigliaro@unipa.it (A.C.); riccardo.alessandro@unipa.it (R.A.); 4 Istituto per la Ricerca e l’Innovazione Biomedica (IRIB), 90133 Palermo, Italy

**Keywords:** osteoarthritis, microRNAs, osteoblasts, chondrocytes, SP1, CX43

## Abstract

Osteoarthritis (OA) is a degenerative bone disease that involved micro and macro-environment of joints. To date, there are no radical curative treatments for OA and novel therapies are mandatory. Recent evidence suggests the role of miRNAs in OA progression. In our previous studies, we demonstrated the role of miR-31-5p and miR-33a families in different bone regeneration signaling. Here, we investigated the role of miR-31-5p and miR-33a-5p in OA progression. A different expression of miR-31-5p and miR-33a-5p into osteoblasts and chondrocytes isolated from joint tissues of OA patients classified in based on different Kellgren and Lawrence (KL) grading was highlighted; and through a bioinformatic approach the common miRNAs target Specificity proteins (Sp1) were identified. Sp1 regulates the expression of gap junction protein Connexin43 (Cx43), which in OA drives the modification of (*i*) osteoblasts and chondrocytes genes expression, (*ii*) joint inflammation cytokines releases and (*iii*) cell functions. Concerning this, thanks to gain and loss of function studies, the possible role of Sp1 as a modulator of CX43 expression through miR-31-5p and miR-33a-5p action was also evaluated. Finally, we hypothesize that both miRNAs cooperate to modulate the expression of SP1 in osteoblasts and chondrocytes and interfering, consequently, with CX43 expression, and they might be further investigated as new possible biomarkers for OA.

## 1. Introduction

Osteoarthritis (OA) is a common degenerative joint disease characterized by cartilage degradation, synovitis, subchondral bone sclerosis and osteophyte formation [1]. OA etiology is complex and multifactorial, including biological and biomechanical factors, micro and macro environments, pathogenetic factors such as obesity, joint trauma, joint infection, previous rheumatoid arthritis, muscle weakness, metabolic disorders, disorders of bone turnover and genetics [2]. These factors act alone or in synergy to initiate a cascade of pathophysiological intra-articular reactions, leading to the i) altered activation of the synoviocytes, ii) activation of innate immune non-antigen specific response with the subsequently release of various inflammatory cytokines, and iii) the presence of cell debris and the disruption of the collagenous fibrillar network inside the joint [3]. The response of the bone cells to these different signals leads to a lower regenerative response of the osteoblasts and to an increase in the maturation of the osteoclasts, determining the lower efficiency of the osseointegration of the prosthesis [4]. For these reasons, OA patients suffer from persistent pain, stiffness and disability.

Conventional treatment includes exercise, physical therapy, life-style changes and pain medications, but no radical curative treatments are available and novel therapies for OA are urgently required. The development of cell therapies, especially with the use of mesenchymal stromal cells (MSCs), for the treatment of joint cartilage lesions or worsening of confirmed OA, have proved promising [5]. In recent years, many studies have focused on the possible roles that some miRNAs involved in musculoskeletal processes could have as predictive or prognostic biomarkers of OA and prosthesis osteointegration [6].

MiRNAs are attractive candidates as multifunctional regulators of bone signaling and have been investigated as new biomarkers of bone disease or bone regeneration. In our recent studies, different miRNAs involved in bone regeneration were identified, such as: (i) miRNA-675-5p, a modulator of HIF-1α and Wnt/b-catenin signalings in hMSCs [7]; (ii) miRNA-31-5p, a mechanosensitive-miRNA involved in hMSCs hypoxia response [8]; and miR-33a family miRNAs regulating YAP/TAZ expression during hMSCs osteoblast differentiation [9].

Concerning the role of miRNAs as biomarkers of OA, the following aspects have been highlighted: (i) miRNA expression in osteoporosis patients [10]; (ii) miR-140-3p, miR671-3p and miR-33b-3p as potential biomarkers for the evaluation of OA risk and progression [11]; and (iii) the differential miRNAs expression in synoviocytes isolated by fluid and plasma of OA patients as promising diagnostic biomarkers [6].

During the last few years, evidence has suggested the involvement of a specific protein (Sp)- transcription factor family in OA progression, whose role in osteosarcoma progression has been already investigated by bioinformatic approaches [12]. The essential role of Sp7 (Osterix) for bone development and mineralization has been already investigated through in vitro and in vivo approaches [13,14,15], by means of physiological and pathological models, while the possible role of specific protein (Sp)- transcription factor 1 (Sp1) has been partially understood [16,17]. Indeed, the in vitro studies in osteoblasts [16,18], osteoclasts [19], chondrocytes [20] and BMSCs [17] have suggested the role of Sp1 in bone cell differentiation and formation, regulating for example COL1A1 and Frizzled1 (FZD1) expression and consequently the regulation of osteoblast mineralization [13]. Sp1 regulates the expression of multiple genes, such as nuclear protein and basal transcription factors that showed on their promoter regions’ GC-rich sequences, which are Sp1 binding sites. The polymorphism to Sp1 binding sites in osteoblast genes reduced bone mineral density and increased risk of osteoporotic fracture [13,21,22].

Several in vitro studies showed a new role of Sp1 as regulator of the extracellular matrix and gap-junction proteins. The latter were found to be altered in OA contributing to the development of the disease; this alteration induces a modification in inflammatory response, mechanotrasduction signaling and gene expression through a direct link to Connexin-response CT-rich sequences in the target promoter genes [23]. Connexin-43 (Cx43) is one of the gap-junction targets of Sp1, but the interaction between Sp1 and Cx43 has been not well defined. In bone tissue, CX43 showed a Sp1 binding site in its promoter, favoring an efficient ERK1/2 activation and promoting osteoblast genes expression and functions [24]. In addition, new evidences suggest that CX43 regulates osteoblast differentiation through the additional recruitment of Sp1 to the osteoprotegerin proximal promoter, resulting in robust transcription of anti-osteoclastogenic factor, thus favoring the osteoblast differentiation [25,26]. Recent proteomic evaluations have suggested a direct role of Cx43 in the development of OA, through an enrichment of CX43 interactors in OA samples compared to control samples [27]. In addition, Stains et al., demonstrated the ability of CX43 to alter the recruitment of Sp1/ Sp3 to the promoter of COL1A1 or other genes, modifying their expression, particularly in the tumor model of osteosarcoma [28].

In the present study, the possible involvement of miR-31-5p and miR-33a-5p in the regulation of crosstalk between SP1 and CX43 in OA disease was investigated in vitro to define their prognostic role in OA disease monitoring. Preliminary miRNAs expression was carried out in osteoblasts (OB) and chondrocytes (CH) isolated from waste surgical tissues of hospitalized patients who underwent joint arthroplasty with different Kellgren and Lawrence (KL) grading. Through bioinformatic analysis, it was determined which miRNAs were strongly modulated in isolated cells and the correlation between the common miRNAs target SP-1 and its relative target Cx43 was investigated.

## 2. Results

### 2.1. miRNAs Expression in OA Patients Derived Cells

**Figure 1:**
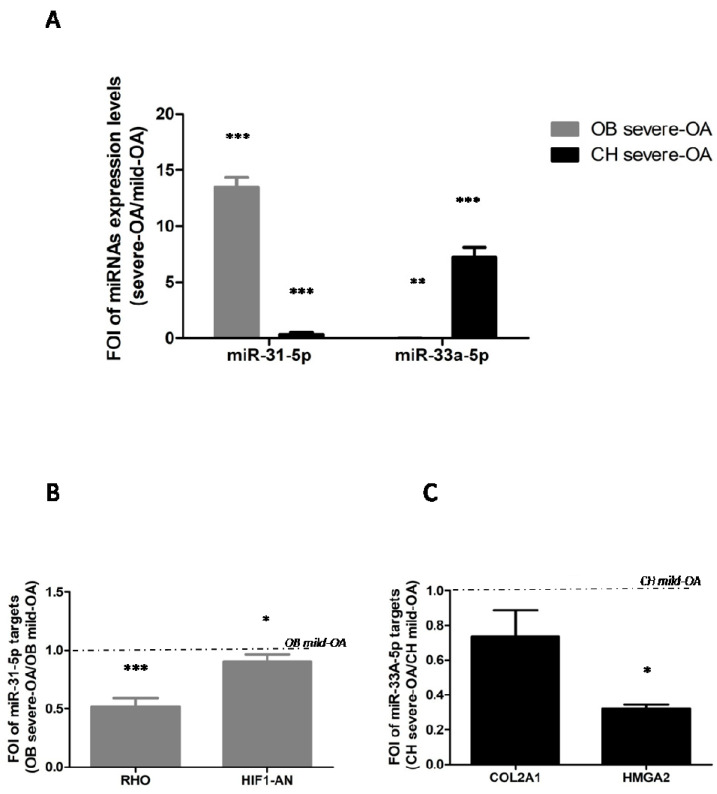
Investigation of osteoblasts (OB) and chondrocytes (CH) isolated by tissue debris of mild OA and severe OA patients through qRT-PCR expression analysis of (**A**) miR-31-5p and miR-33a-5p; (**B**) miR-31-5p OB targets genes: RHO and HIF-1AN; and (**C**) miR-33a-5p CH targets genes: COL2A1 and HMGA2. Quantitative RT-PCR data are expressed as fold of change (FOI) in miRNAs or gene expression (2^−ΔΔCt^) occurred in severe OA-derived cells vs mild OA-derived cells. Data reported were analysed by Student *t* test: *, *p* < 0.05, **, *p* < 0.005, ***, *p* < 0.0005 between experimental group and represented as mean ± SD (*n* = 4).

Starting from our recent evidences about the role of miR-31-5p [8] and miR33a-5p [9] in bone regeneration processes, we investigated the involvement of both miRNAs in mild- and severe OA-derived OB and CH dysfunctions during OA disease. Severe OA derived OB and cells showed a different expression of miR-31-5p and miR-33a-5p levels compared to mild OA-derived cells. Severe OA-derived OB displayed an increase of miR-31-5p expression levels compared to mild OA-derived OB (*p* < 0.0005) and no modulation of miR-33a-5p was found, as shown by the significantly lower FOI values *(p* = 0.04), while severe OA-derived CH showed high levels of miR-33a-5p expression compared to mild OA CH (*p* < 0.0005) and no modulation of miR-31-5p expression was shown again by the significant lower FOI values (*p* < 0.0005) (Figure 1A). To underline the miRNAs specific-action in isolated cells, we investigated of the expression of miRNAs specific targets in: OB-RHO (*p* < 0.0005) and HIF-1AN (*p* = 0.04) for miR-31-5p [8], and CH-HMGA2 (*p* = 0.01) and COL2A1for miR-33a-5p [9] (Figure 1B,C).

### 2.2. Investigation of miRNA31-5p and miR-33a-5p Common Targets

**Figure 2:**
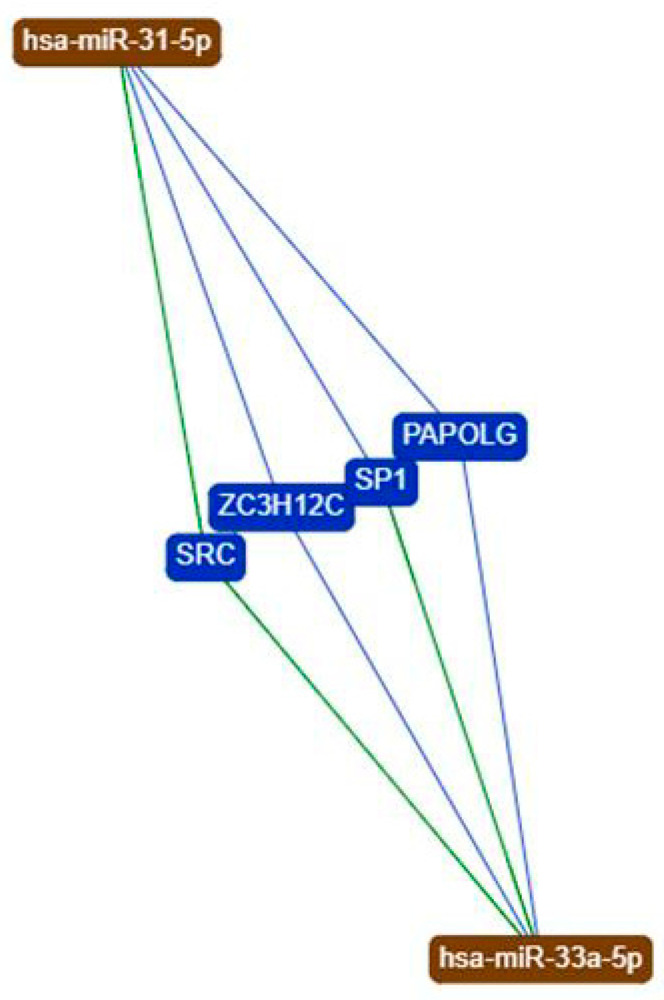
Analysis of miRNAs common target SP1 by the bioinformatics tool miRTargetLink Human. The central node represents Sp1 and other common targets, surrounded by the miRNAs that target Sp1 with strong (green) and weak (blue) evidence.

To investigate the possible common targets between miR-31-5p and miR-33a-5p involved in OA signaling, a bioinformatic analysis of miR-31-5p and miR-33a common predicted targets was performed. Using the bioinformatics tool miRTargetLink Human (https://ccb-web.cs.uni-saarland.de/mirtargetlink/ (accessed on 27 February 2021)) we identified the miRNAs common targets, as shown in the central node of Figure 2. The central node is surrounded by the validated targets with strong (e.g., luciferase assay in green) and weak (e.g., microarray in blue) evidence. Transcription factor SP1 was one of the predicted targets; it showed a strong and weak link with miR-33a-5p and miR-31-5p, respectively, as demonstrated by miRTarBase investigation (http://miRTarBase.mbc.nctu.edu.tw/ (accessed on 27 February 2021)).

### 2.3. Modulation of SP-1 Expression in OA

**Figure 3:**
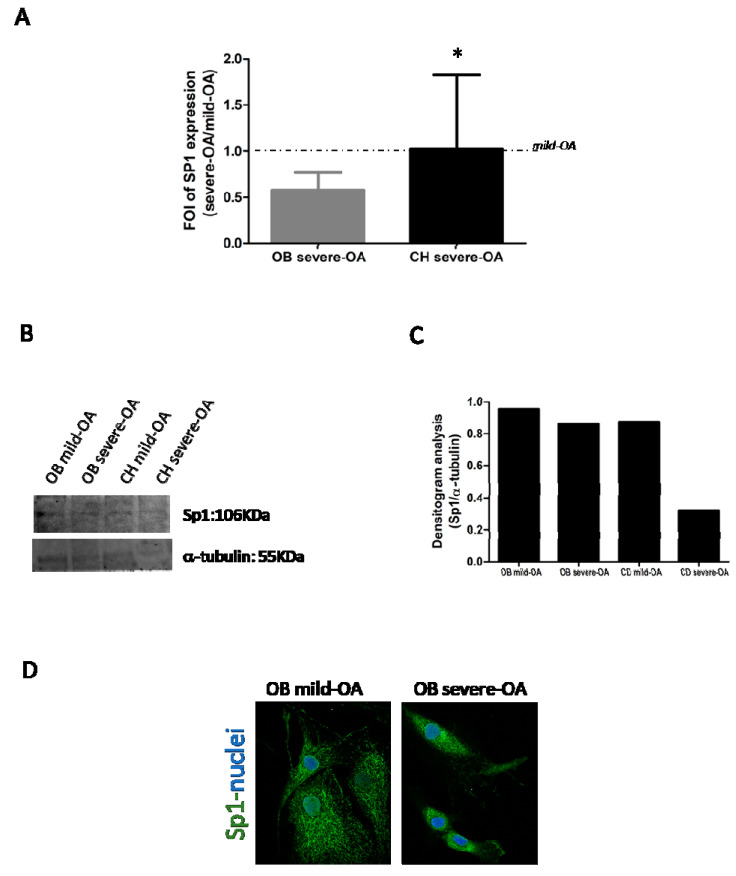
Analysis of SP1 gene and protein expression on osteoblasts (OB) and chondrocytes (CH) derived by patients with mild OA and severe OA. (**A**) qRT-PCR analysis of SP1 gene expression. Data are expressed as fold of change (FOI) in gene expression (2^−ΔΔCt^) occurred in severe OA vs mild OA isolated cells. Data reported were analysed by Student *t* test: *, *p* < 0.05, between experimental group and represented as mean ± SD (*n* = 4). (**B**,**C**) Western blot and densitogram analysis of Sp1 and α-tubulin proteins were performed on total cell extract isolate by primary cells derived by mild- and severe OA groups. (**D**) Confocal analysis of Sp1 protein expression and localization on OB isolated by mild OA and severe OA patients. In green the Sp1 signals while in blue the nuclei localization.

In order to validate the correlation between the miRNAs (miR-31-5p and miR-33a-5p) and SP1, firstly we performed a qRT-PCR and western blot analysis of mild OA and severe OA-derived OB and CH cells. Data in Figure 3 revealed lower levels of Sp1 mRNA (A) and protein (B) in severe OA samples thus supporting an inhibitor role of the miR-31-5p and miR-33a-5p on Sp1 expression. Confocal analysis performed on mild OA and severe OA-derived OB showed a reduction in nuclear localization thus confirming data obtained with western blot (Figure 3D).

**Figure 4:**
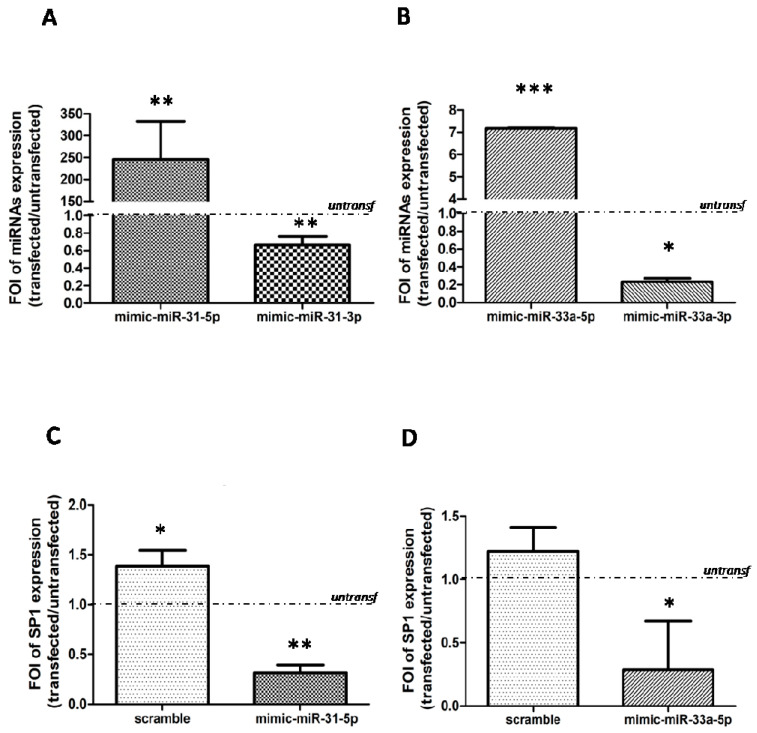
Study of SP1 gene expression modulation by gain and loss of function assays. Osteoblasts (OB) and chondrocytes (CH) isolated by mild OA patients (mild OA) after 24 h of transfection with: miR-31-5p mimic and miR-31-3p mimic for OB cells, with miR-33a-5p mimic and miR-33a-3p mimic for CH cells and relatives scrambles. Cells were analyzed for the gene expression modulation of SP1 by qRT-PCR analysis. (**A**) The efficiency of miR-31-5p mimic and miR-31-3p mimic and relatives scrambles transfection were evaluated in transfected mild OA-derived OB versus untransfected ones. (**B**) The efficiency of miR-33a-5p mimic and miR-33a-3p and relatives scrambles transfection were evaluated in transfected mild OA-derived CH versus untransfected ones. SP1 gene expression evaluated in (**C**) OB isolated from mild OA patients transfected with mimic-31-5p and negative scramble, in (**D**) CH isolated from mild OA patients transfected with mimic-33a-5p and negative scramble. Quantitative RT-PCR data are expressed as fold of change (FOI) in miRNAs or gene expression (2^−ΔΔCt^) occurred in transfected mild OA isolated cells vs untransfected ones. Data reported were analysed by Student *t* test: *, *p* < 0.05, **, *p* < 0.005, ***, *p* < 0.0005 between experimental group and represented as mean ± SD (*n* = 4).

To further demonstrate our hypothesis, we performed a gain and loss of function studies on OB and CH derived by mild OA and severe OA patients. Firstly, we over-expressed miR-31-5p (*p* = 0.0013) and miR-31-3p (*p* = 0.006), and miR-33a-5p (*p* < 0.0005), miR-33a-3p (*p* = 0.02), respectively in OB (Figure 4A) and CH cells (Figure 4B) derived by mild OA group. QRT-PCR analysis revealed that the over-expression of miR-31-5p mimic into mild OA derived OB group induces the downregulation of SP1 mRNAs compared to untransfected cells (Figure 4C, *p* < 0.0005). In the same manner, the over-expression of miR-33a-5p mimic into mild OA-derived CH group induces the downregulation of SP1 mRNAs compared to untransfected ones (Figure 4D, *p* = 0.03).

**Figure 5:**
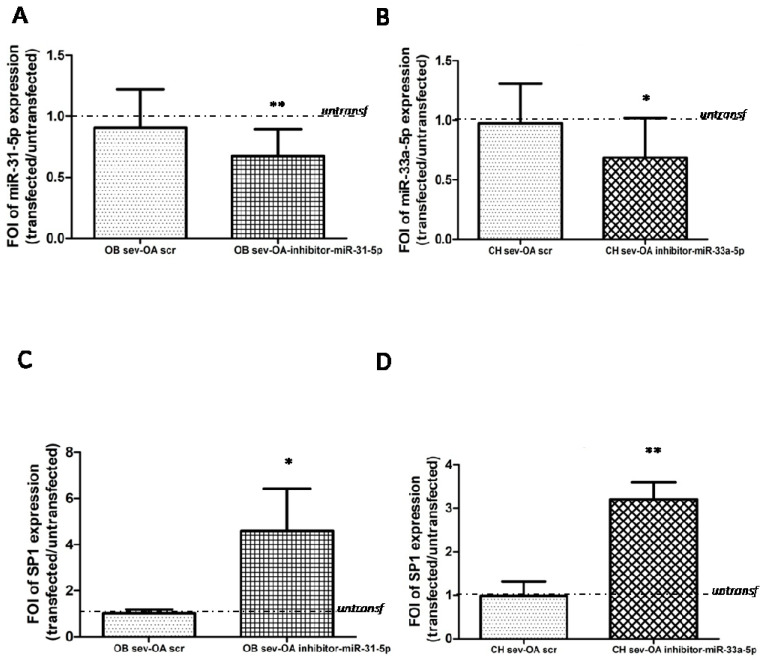
Study of SP1 gene expression modulation by gain and loss of function assays. Osteoblasts (OB) and chondrocytes (CH) isolated by OA patients after 24 h of transfection with miR-31-5p inhibitor for OB cells and with miR-33a-5p inhibitor for CH cells and relatives scrambles were analyzed for the gene expression of SP1 by qRT-PCR analysis. (**A**) The efficiency of miR-31-5p inhibitor and relative scrambles transfection was evaluated in transfected severe OA-derived OB versus untransfected ones. (**B**) The efficiency of miR-33a-5p inhibitor and relatives scramble transfection was evaluated in transfected severe OA-derived CH versus untransfected ones. SP1 gene expression evaluated in (**C**) OB isolated from severe OA patients transfected with miR31-5p inhibitor and negative scramble, and in (**D**) CH isolated from severe OA patients transfected with miR-33a-5p inhibitor and negative scramble. Quantitative RT-PCR data are expressed as fold of change (FOI) in miRNAs or gene expression (2^−ΔΔCt^) occurred in transfected OA derived cells vs untransfected ones. Data reported were analysed by Student *t* test: *, *p* < 0.05, **, *p* < 0.005, between experimental group and represented as mean ± SD (*n* = 4).

To support our hypothesis we over-expressed the inhibitor of miR-31-5p in severe OA-derived OB, which induced the increase of SP1 mRNAs levels compared to untransfected severe OA-derived OB (Figure 5A, *p* = 0.006; Figure 5C, *p* = 0.02). In the same manner, the over-expression of miR-33a-5p inhibitor into CH severe OA cells induced the increase of SP1 mRNAs levels compared to CH severe OA untransfected cells (Figure 5B, *p* = 0.01; Figure 5D, *p* = 0.001).

### 2.4. Connexin 43 Expression in OA Derived Cells

**Figure 6:**
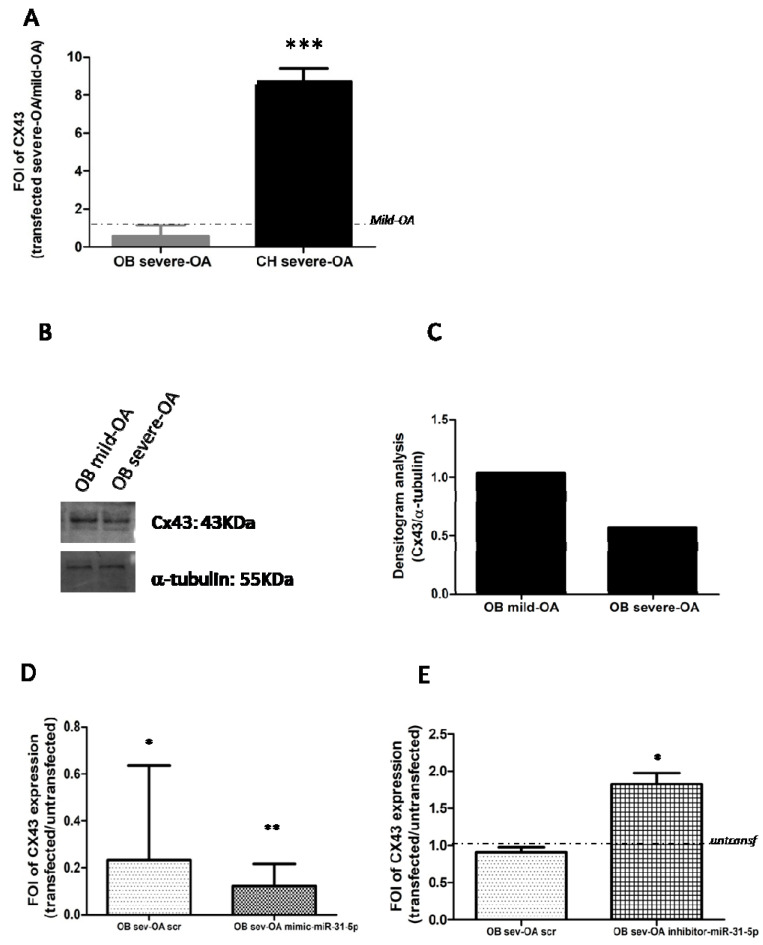
Analysis of CX43 gene and protein expression on osteoblasts (OB) and chondrocytes (CH) derived by patients with severe and mild grade OA. (**A**) qRT-PCR analysis of CX43 gene expression. (**B**,**C**) Western blot and densitogram analysis of Cx43 and α-tubulin proteins were performed on total cell extract isolated by OB primary cells. (**D**) qRT-PCR analysis of CX43 gene expression in OB isolated by mild OA patients and transfected with miR-31-5p mimic and relative scramble. (**E**) CX43 gene expression analysis by qRT-PCR in OB isolated by severe OA patients and transfected with miR-31-5p inhibitor and relative scramble. qRT-PCR data are expressed as fold of change (FOI) in gene expression (2^−ΔΔCt^) occurred in transfected vs untransfected cells. Data reported were analysed by Student *t* test: *, *p* < 0.05, **, *p* < 0.005, ***, *p* < 0.0005 between experimental group and represented as mean ± SD (*n* = 4).

It is reported that Sp1 regulates the Connexin 43 gene promoter (CX43) in physiological and pathological conditions. To investigate the regulative role of Sp1 on Cx43 gene promoter^13^, we performed qRT-PCR and Western Blot analysis of CX43 in OB and CH cells derived by mild- and severe OA patients. As showed in Figure 6, CX43 was down-regulated in severe OA-derived OB in terms of gene (Figure 6A) and protein levels (Figure 6B,C), while its gene was up-regulated in severe OA-derived CH (*p* < 0.0005) compared to mild OA cells (Figure 6A). These data suggest that miR-31-5p probably regulates the expression of CX43 mRNAs in severe OA-derived OB. The modulation of Cx43 expression to mild and severe OA-derived OB was confirmed by confocal analysis (Figure S1).

Through a gain and loss of function study on OB derived by severe and mild patients these data were verified. We over-expressed miR-31-5p in mild OA-derived OB and, as expected, qRT-PCR analysis revealed a down-regulation of CX43 mRNAs levels (Figure 6D) in transfected mild OA-derived OB with mimic of miR-31-5p (*p* = 0.08) compared to untransfected ones (*p* = 0.003); on the contrary, the over-expression of miR-31-5p inhibitor induced an increase of CX43 expression in the severe OA-derived OB group compared to untransfected ones (Figure 6E; *p* = 0.01).

To understand the difference in the regulation of CX43 gene expression between both primary cells, we evaluated the expression of CX43 in hMSCs. The supplementary figure (Figure S2) shows the differences of Cx43 protein expression in hMSCs transfected with miR-31-5p and miR-33-5p mimic compared to relative untransfected cells. The hMSCs after miR-31-5p over-expression showed a decrease in Cx43 expression compared to scramble cells; while after miR-33a-5p over-expression showed an increase of Cx43 protein expression respect scramble cells. Surprisingly, SP1 mRNA analysis revealed no significant down-regulation of its expression in term of mRNA after both transfections.

## 3. Discussion

During the last few years the molecular aspects of OA development and progression have been deeply investigated with the aim to find alternatives to current treatments that until now permitted only the controlling of joint pain and inflammation. MiRNAs have been noted not only as key molecules in intracellular regulatory networks for gene expression [29], but also as biomarkers for various pathological conditions [30,31,32]. Today, several miRNAs have been found to display an aberrant expression level in OA [10,11] and thus it would be very interesting to understand how they act in OA pathogenesis, making them useful as biomarkers or therapeutic targets for OA diagnosis, monitoring and treatment.

In the present study we investigated the role of miR-31-5p and miR-33a-5p in OA disease and progression. Previously, it was shown that miR-31-5p displayed a central role during bone regeneration, modulating the expression of cytoskeletal proteins and hypoxia signaling [8]. Moreover, miR-33a-5p showed a different expression in hMSCs and primary OB regulating the expression and activation of YAP/TAZ signaling^9^ during hMSCs osteoblast differentiation process, which were also modified in OA. In this preliminary investigation, we evaluated the expression of miR-31-5p and miR-33a-5p in OB and CH cells isolated by patients hospitalized for surgery of endo- or arthroplasty for OA (Group severe OA), and for surgery for joint fractures (i.e., femoral neck fractures) requiring joint arthroplasty (Group mild OA). First of all, we noted how OB and CH cells isolated by severe OA patients expressed miR-31-5p and miR-33a-5p, respectively (Figure 1), as confirmed by the specific regulation of their miR-targets. Secondly, the bioinformatic analysis highlighted that both miRNAs showed some common gene targets; in particular we focused our attention on specific protein 1 (SP1) (Figure 2) that displayed a strong role in the regulation of bone functionality [12,13,15,17,18,22,28] and OA disease compared to other common targets identified.

Sp1 is a common transcription factor and plays an important role in cellular growth, bone differentiation and function and chondrocytes actions. It regulates bone cell differentiation and activity by controlling the levels of transforming growth factor β type I receptor (TGFβ-RI) and regulation of RUNX2 expression during osteogenesis, and targeting FZD-1 during the differentiation process [12]. Increased Sp1 binding to the type II collagen gene (COL2A1) promoter is required for the stimulation of COL2A1 gene expression by 17β-estradiol in differentiated and dedifferentiated rabbit chondrocytes [20,21]. In our opinion, these are some reasons because Sp1 might be considered strongly correlated to OA. Current data confirmed the role of SP1 in OA progression, because severe OA-derived cells displayed a downregulation of SP1 mRNAs and protein expression compared to each mild OA ones; in particular, in severe OA-derived OB cells we observed a strongly reduction of SP1 (Figure 3). The gain and loss of function studies highlighted the involvement of both miRNAs into SP1 expression (Figure 4 and Figure 5). The mild OA-derived OB overexpressing miR-31-5p showed a downregulation of SP1 mRNAs, while the overexpression of miR-31-5p inhibitor in severe OA-derived OB induced an upregulation of SP1 mRNA expression. In the same manner, mild OA-derived CH overexpressing miR-33a-5p confirmed a downregulation of SP1 mRNA expression, and the overexpression of miR-33a-5p inhibitor in severe OA-derived CH induced an upregulation of SP1 mRNA expression.

Recent studies demonstrated a direct link between SP1 and Connexin 43 (Cx43), one of the new actors of OA disease [33,34]. Cx43 is a component of gap junctions and plays a critical functional role, permitting a direct cellular communication through the intercellular exchange of ions, small RNAs, nutrients and second messengers [26,27,35]. The Cx43 protein is overexpressed in several human diseases and inflammation processes and in articular cartilage from patients with OA [24]. An increase in the level of Cx43 is known to alter gene expression, cell signaling, growth and cell proliferation [24,33]. A recent proteomic analysis revealed several new Cx43 dependent pathways strongly involved in OA disease, such as: glucose metabolism, calcium flux, etc [27].

In our experimental setup, we observed a downregulation of CX43 in severe OA-derived OB and an increase in severe OA-derived CH compared to mild OA (Figure 6). These data suggest that the regulation mediated by SP1 to CX43 promoter is present in severe OA-derived OB cells, while severe OA-derived CH is probably regulated by others interactor proteins [27]. Again, gain- and loss-of-function studies confirmed present results. OB obtained by mild OA patients after transfection with miR-31-5p mimic showed a downregulation of CX43 compared to untransfected ones, while OB collected by severe OA patients overexpressing miR-31-5p inhibitor showed an increase of CX43 expression compared to untransfected ones. These data provide evidence that the regulation of CX43 mRNA in OB is probably mediated by Sp1 protein expression during OA progression. Notably, the overexpression or inhibition of miR-33a-5p in mild or severe OA-derived CH did not alter the expression of CX43 compared to relative scramble groups.

Finally, to understand the possible link between miR-31-5p or miR-33a-5p and CX43 expression in the bone microenvironment, we decided to investigate other cell populations involved in OA and bone regeneration. We evaluated the modulation of CX43 into hMSCs commercial cell lines after miR-31-5p and miR-33a-5p expression. Also, in the hMSCs model, miR-31-5p overexpression induced the downregulation of Cx43 protein, while miR-33a-5p overexpression did not modify the expression of Cx43 (Figure S2A). Concerning the expression of SP1, hMSCs displayed no significant downregulation of SP1 mRNA expression after both miRNAs-transfection (Figure S2B). However, our results suggest that in OA disease miR-31-5p and miR-33a-5p displayed the same control of SP1 expression, but differently regulated SP1 target CX-43.

**Figure 7:**
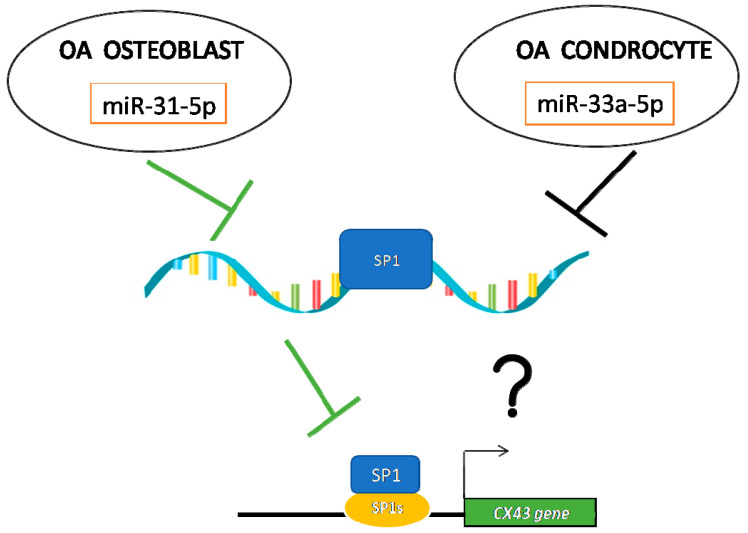
Our hypothesis of the molecular mechanism of miR-31-5p and miR-33a-5p on SP1/CX43 regulations in OA condition.

From the evidence obtained, we hypothesize a different mechanism of action of both miRNAs in OB and CH derived by OA samples (Figure 7).

During OA, osteoblast cells undergo a cytoskeleton modification through not only miR-31-5p up-regulation, as demonstrated in our previous study [8], but also thanks the down-regulation of SP1 and consequently alteration in OB differentiation and functionality and CX43 expression. Regarding chondrocytes, they presented less down-regulation induced by miR-33a-5p to SP1 expression compared to the OB severe OA-group and no modification was identified in CX43 expression. In our opinion, these data suggest a role of a new interactor protein in the CX43 promoter gene (data no shown). Regarding this, recent evidences suggest the role of EMT proteins in CX43 expression and the role of miR-33a-5p as a modulator of EMT markers in bone regeneration. Even though a limit of the current study might be the few chondrocyte and osteoblast donors, the significance and particularity of these first preliminary results make us confident that further investigations such as proteomic analysis on isolated cells of all enrolled patients (ongoing clinical study enrolling a total of *n* = 5 patients per mild and severe OA) will confirm the current results and allow us to better understand the role of miR-33a-5p in CX43 expression and highlight the role of miR-31-5p in the OA microenvironment during OA progression.

## 4. Material and Methods

### 4.1. Cell Cultures and Reagents

Osteoblasts (OB) and chondrocytes (CH) were isolated from waste surgical joint tissues (Protocol ID: CE AVEC 287/2018/Sper/IOR) of patients aged >40 years hospitalized for surgery of i) endo- or arthroplasty for OA with Kellgren and Lawrence (KL) grading >2 (Group severe OA: *n* = 2 patients); or ii) joint fractures (e.g., femoral neck fractures) requiring the implantation of a joint prosthesis that showed KL grading ≤2 (Group mild OA: *n* = 2 patients). Table 1 reports the demographic and clinical data of selected patients. OB and CH were isolated according to appropriate protocol^6, 24^, and maintained in culture in specific differentiated mediums: OBs in Osteoblast medium (Osteoblast Growth Medium—iX Cells Biotechnologies MD-0054) and CHs in chondrocyte medium (Human Chondrocyte Media—Cell Applications.MD.411–500).

Commercially available human mesenchymal stromal cells (hMSCs-Lonza, Walkersville, MD, USA) were cultured in Mesenchymal Stem Cell Growth Medium (MSCGM™ Bullet Kit, Lonza, Walkersville, MD, USA). Culture medium was changed every three days and cells were split at 70–80% of confluence using Stem Pro Accutase (Gibco by Life Technologies Italia, Monza, Italy). All cells were maintained in culture in a humidified atmosphere of 5% CO_2_ at 37 °C until the 3rd passages; then, the cells were plated to perform the following assays.

### 4.2. Cell Transfection

For cell transfection, Attractene Transfection Reagent (cat. number 1051531, Qiagen Srl, Milan, Italy) was used following the manufacturer’s indication. Briefly, cells seeded at 150,000 cells/cm^2^ were transfected with 15 pmoles/mL of: has-mir-31-5p mimic (4464066-MC11465, mirVana miRNA mimics Life Technologies Italia, Monza, Italy); has-mir-31-3p mimic (4464066-MC12887, mirVana miRNA mimics-Life Technologies, Monza, Italy); has-miR-31-5p inhibitor (4464084-MH11465, mirVana miRNA inhibitors-Life technologies Italia, Monza, Italy); hsa-mir-33a-5p mimic (4464066-MC12410, mirVana miRNA mimics-Life Technologies, Monza, Italy); hsa-mir-33a-3p mimic (4464066-MC12607, mirVana miRNA mimicsLife Technologies, Monza, Italy); hsa-mir-33a-5p inhibitor (4464084-MH12410, mirVana miRNA inhibitors -Life Technologies, Monza, Italy); and scrambled negative controls (4464058, mirVana negative control Life Technologies, Monza, Italy) for 24h. These last are negative controls of tested miRNA mimics and the target gene expression from negative control-transfected samples are used as a baseline values for the evaluation of the effect of the control and experimental miRNA mimic on target gene expression. All transfections with miRNAs mimic and relative scrambles (alias negative control) were performed on cells derived by mild OA patients (identified as OB or CH mild OA), while the transfection with miRNAs inhibitors described above and relative scrambles were performed on cells isolated by severe OA patients (identified as OB or CH severe OA). At each experimental time the cells are processed for the following assays.

### 4.3. RNA Extraction and Real-Time PCR

Total RNA was extracted using the commercially available illustraRNAspin Mini Isolation Kit (GE Healthcare, Milan, Italy), according to the manufacturer’s instructions. RNA was reverse transcribed to cDNA using the High Capacity cDNA Reverse Transcription Kit (Applied Biosystems, ThermoFisher Scientific, Monza Italy). Quantitative RT-PCR (qRT-PCR) analysis was performed in duplicate for each data point, using custom made primers (Invitrogen, Life Technologies Italia, Monza, Italy) (Table 2). The mean threshold cycle was used for the calculation of relative expression using the Livak method against ACTB [37,38].

For miRNA expression, 250 ng of RNA was reverse transcribed according to the manufacturer’s instructions (cat. number 4366596, TaqMan MicroRNA Reverse Transcription, Applied Biosystems, ThermoFisher Scientific, Monza, Italy). Taqman probes were used to analyze miR-31-5p (4427975-ID002279, Applied Biosystem, ThermoFisher Scientific), miR31-3p (4427975-ID002113, Applied Biosystem, ThermoFisher Scientific), miR-33a-5p (4427975-ID002279, Applied Biosystem, ThermoFisher Scientific), and miR-33a-3p (4427975-ID002113, Applied Biosystem, ThermoFisher Scientific) Changes in the target miRNA content was calculated in relation to the housekeeping *RNU6-1* “RNA, U6 small nuclear 1” (4427975 Applied Biosystem, ThermoFisher Scientific).

### 4.4. Western Blot Analysis

SDS-PAGE and Western blotting (WB) were performed according to standard protocols. Briefly, after transfection, cells were lysed in lysis buffer containing 15 mM Tris/HCl pH 7.5, 120 mM NaCl, 25 mM KCl, 1 mM EDTA, 0.5% Triton ×100, Halt Protease Inhibitor Single-Use cocktail (100×, Fisher Scientific Italia, Rodano, Italy). Whole lysate (15 µg per lane) was separated using 4–12% NovexBis-Tris SDS-acrylamide gels (Invitrogen, Life Technologies Italia, Monza, Italy), electro-transferred on nitrocellulose membranes (Bio-Rad Laboratories Srl, Segrate, Milan, Italy) and immunoblotted with the appropriate antibodies. Antibodies against the following proteins were used: Sp1(Sp1 (E-3) Antibody, sc-17824), Cx43(connexin 43 (F-7) Antibody, sc-271837), α-Tubulin (monoclonal anti-α-Tubulin (TU-02), sc8035, Santa Cruz Biotechnology INC.,). All secondary antibodies were obtained from Fisher Scientific Italia. Immunofluorescence was detected using a CCD high resolution and high sensitivity detection technology (ChemiDoc™ XRS+ System, Bio-Rad Laboratories Srl, Segrate (MI), Italy).

### 4.5. Immunofluorescence Analysis

Immunocytochemistry was carried out on osteoblast cells (OB mild OA and OB severe OA) transfected with scramble and mimic for 24 h, and stained with Sp1 (Sp1 (E-3) Antibody, sc-17824) and Cx43 (connexin 43 (F-7) Antibody, sc-271837) and the secondary antibody Alexa-Fluor 488 from Molecular Probes. The nuclei were stained with NucRed^®^ Live 647 (cat. number: R37106, Life Technologies), and preparations were analyzed by confocal laser microscopy (Eclipse A1+ Ti, Nikon Instruments SpA, Campi Bisenzio, Italy).

### 4.6. Statistical Analysis

Statistical analysis was done by using R software v.4.0.3. [37]. Data are reported as mean ± SD (*n* = 4) at a significant level of *p* < 0.05. Student t test was used to compare data between groups.

## 5. Conclusions

In conclusion, the current findings provide insightful knowledge on the significant role of miR-31-5p and miR-33a-5p in OA. The understanding of their molecular mechanisms in regulating SP1 expression might be pivotal to better clarifying osteoblast dysfunctions and chondrocytes modifications during the OA pathophysiological processes. In the current study, the crosstalk between SP1 and CX43 signaling, mediated by the probable regulation of miR-31-5p and miR-33a-5p, respectively, in osteoblasts and chondrocytes, has been preliminary elucidated. Findings provided novel evidence on miR-31-5p and miR-33a-5p as new biomarkers for identification of different OA grades, and eventually as possible actors of personalized gene therapies for treating OA disorders.

## Figures and Tables

**Table 1:** Demographic and clinical data of selected patients.

	Group Severe OA		Group Mild OA	
	Woman	Man	Woman	Man
Age (yrs)	74	64	62	63
Weight (kg)	62	58	64	68
BMI (kg/m²)	25.8	24.1	26.0	23.8
Kellgren and Lawrence grading [36]	4	4	2	2
Other pathologies	-	-	-	Diverticulosis
Therapy	Atenolol 50 mg/die	-	-	Olmesartan Medoxomil/Amlodipine, 20/5 mg/die
WBC (×10³/μL)	6.76	6.85	5.45	8.42
CRP (mg/L)	0.17	0.34	0.30	0.20

**Table 2:** List of gene primers used to study gene expression profiling. Their expression was normalized to the β-actin housekeeping gene.

Gene	Primer Forward	Primer Reverse
*RHOA* “Transforming protein RhoA”	GAAAACCGGTGAATCTGCGC	AGAACACATCTGTTTGCGGA
*HIF-1AN* “Hypoxia-inducible factor 1-alpha inhibitor”	TGGGGGCAGCTTACCTCTAA	TGGGTAGAGGCACTCGAAC
*HMGA-2* “High mobility group AT-hook 2”	GCGCCTCAGAAGAGAGGAC	GTCTTCCCCTGGGTCTCTTAG
*COL2A1* “Collagen type II alpha 1 chain”	CCTGGCAAAGATGGTGAGACAG	CCTGGTTTTCCACCTTCACCTG
*SP-1* ”Specific protein 1”	GCCTCCAGACCATTAACCTCAGT	GCTCCATGATCACCTGGGGCAT
*CX-43* “Connexin43”	GAACTCAAGGTTGCCCAAAC	TTAGAGATGGTGCTTCCCG
**Reference Gene**		
*ACTB* “Beta-Actin”	ATCAAGATCATTGCTCCTCCTGA	CTGCTTGCTGATCCACATCTG

## Data Availability

The data presented in this study are available on request from the corresponding author. The data are not publicly available due to non-completion of the clinical study.

## Supplementary Materials

Supplementary materials are available online at https://www.mdpi.com/1422-0067/22/5/2471/s1.
