# Supplementary material for: How miR-31-5p and miR-33a-5p Regulates SP1/CX43 Expression in Osteoarthritis Disease: Preliminary Insights
Source: Int J Mol Sci. 2021 Feb 28;22(5):2471. doi: 10.3390/ijms22052471 (PMC7957523; doi:10.3390/ijms22052471)
Supplement: Supplementary file 1 [file ijms-22-02471-s001.pdf]

Supplementary Figure 1

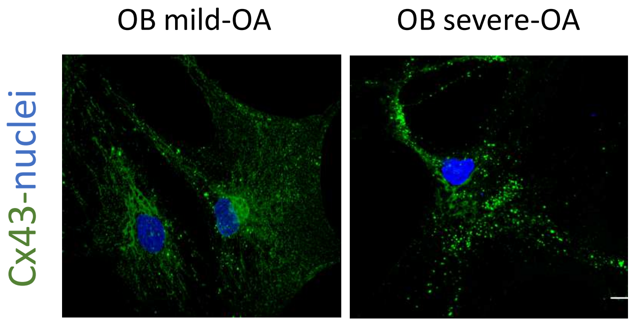

Figure 1S: Confocal analysis of Cx43 protein expression and localization on OB isolated by OA patients (mild and severe grade); in green the Cx43 signals, while in blue the nuclei localization.

Supplementary Figure 2

A

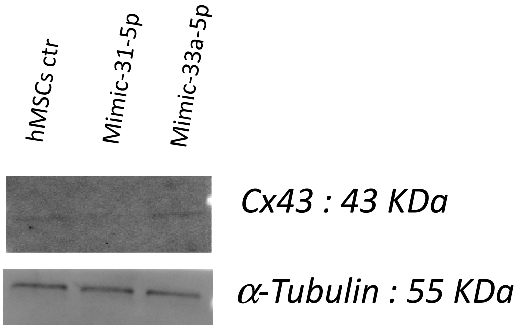

B

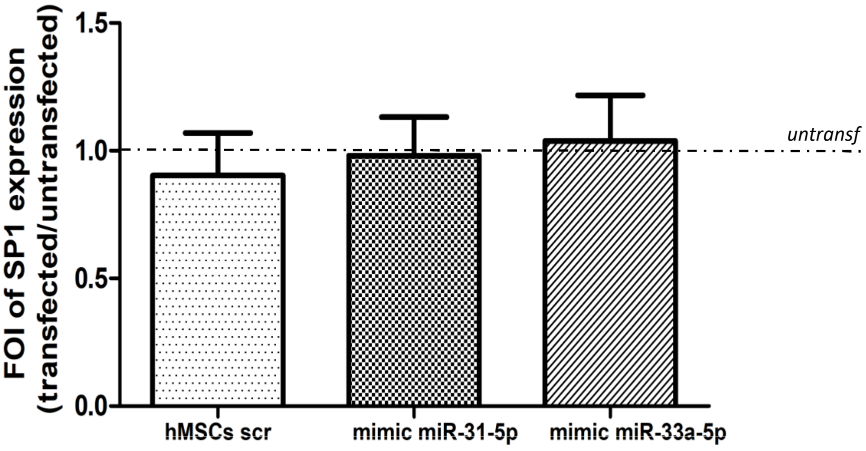

Figure 2S: Preliminary investigation on Cx43 and SP1 expression into hMSCs.

A) Evaluation of Cx43 and a-tubulin expression on hMSCs transfected with scramble, miR-31-5p mimic and miR-33a-5p mimic.

B) qRT-PCR analysis of SP1 expression on hMSCs transfected with scramble, miR-31-5p mimic and miR-33a-5p mimic Data are represented as fold of change (FOI) in gene expression ($2^{-\Delta\Delta Ct}$) occurred in transfected cells *vs* untransfected cells. Student t test: $p<0.05$, **, $p<0.005$, ***, $p<0.0005$ between experimental group.
